# Mechanical and adhesive properties of monolithic zirconia and their clinical implications: a narrative review

**DOI:** 10.1007/s44445-026-00136-0

**Published:** 2026-05-02

**Authors:** Areej Ata Abdulgader

**Affiliations:** Department of Restorative Dental Science, King Abdullah Bin Abdulaziz University Hospital, Princess Nourah Bint Abdulrahman University, 13412 Riyadh, Saudi Arabia

**Keywords:** Dental ceramics, Zirconia, Monolithic zirconia, Yttria-stabilized zirconia, Flexural strength, Adhesion, Surface treatment, Zirconia primer, Resin cement, Resin monomers

## Abstract

Monolithic zirconia has become increasingly popular in clinical dentistry as an indirect restorative material fabricated using computer-aided design/computer-aided manufacturing (CAD/CAM) technology. It is widely used due to its favorable combination of mechanical strength, aesthetic potential, and biocompatibility. Its monolithic design reduces the risk of veneer chipping, thereby improving restoration longevity. To narratively review the mechanical and adhesive properties of monolithic zirconia and discuss their clinical implications. This narrative review was based on a comprehensive, non-systematic literature search conducted using PubMed/MEDLINE, Scopus, and Web of Science. English-language publications addressing monolithic zirconia, mechanical behavior, surface treatments, adhesive strategies, and clinical performance were considered. Additional studies were identified through manual screening of reference lists. Study selection was guided by relevance to the review topic rather than predefined inclusion or exclusion criteria. Monolithic zirconia demonstrates high flexural strength and fracture toughness, supporting its use in posterior load-bearing restorations. However, direct exposure to the oral environment may promote low-temperature degradation (LTD), potentially affecting long-term mechanical stability. Despite improvements in translucency, aesthetic performance remains a consideration. Adhesive durability depends largely on appropriate surface conditioning and the use of functional primers, particularly those containing 10-methacryloyloxydecyl dihydrogen phosphate (MDP), which enhance chemical bonding to zirconia. Monolithic zirconia offers a reliable balance between strength and clinical durability. Nevertheless, its long-term performance is influenced by environmental exposure and adhesive protocols. Further research is needed to optimize the resin–zirconia interface while maintaining both mechanical reliability and aesthetic outcomes.

## Introduction

Dental ceramics are materials commonly used to restore or cover missing tooth structure. Historically, using dental ceramic materials alone was limited to the front teeth; however, nowadays, monolithic lithium disilicate and zirconia materials are widely used for both front and back teeth (Helvey 2013). This is because of their aesthetic qualities and high biocompatibility, which have made all-ceramic materials successful alternatives to metal-based restorations (Blatz et al. 2022). Dental ceramics can be classified by several ways**;** including fusing temperature, microstructure (leucite reinforced glass ceramic, alumina based ceramic; feldspathic porcelain, lithium disilicate reinforced glass ceramic, and zirconia), composition (predominantly glass and polycrystalline), processing method (powder/liquid building, slip casting, hot-pressed ceramic, and CAD/CAM material), translucency, fracture resistance, and abrasiveness (Helvey 2013). The longevity of dental ceramic restorations depends primarily on the strength and reliability of the resin-ceramic bond. The bonding protocol is influenced by both the composition and classification of dental ceramics, and generally includes specific surface treatment steps for the restorations (Blatz et al. 2022). Zirconia ceramic materials are widely used in dental practice due to their mechanical strength and biocompatibility. Recently, advancements in zirconia have enhanced its translucency, thereby broadening its range of dental applications. Nevertheless, increased translucency has raised concerns about mechanical strength, long-term durability, and bonding effectiveness, especially after wear or surface treatments. Although numerous laboratory and clinical studies have investigated these challenges, the results remain inconsistent regarding strength, longevity, and optimal bonding protocols. This review synthesizes current research on the strength and bonding of monolithic zirconia, with particular emphasis on material composition, preparation techniques, and long-term performance. The objective is to provide clear, evidence-based recommendations to help dental practitioners make informed decisions about the use of zirconia in restorative procedures.

## Background

### Search strategy

A comprehensive but non-systematic literature search was conducted to identify relevant publications addressing the mechanical and adhesive properties of monolithic zirconia and their clinical implications. Electronic searches were performed using major scientific databases, including PubMed/MEDLINE, Scopus, and Web of Science. Additional articles were identified through manual screening of reference lists of relevant reviews and original studies.

The search focused on English-language publications without strict restrictions on publication date to capture both foundational and recent evidence. Keywords and combinations of terms related to monolithic zirconia, mechanical properties, adhesive behavior, surface treatments, and clinical performance were used. Study selection was guided by relevance to the scope of the review rather than by predefined inclusion or exclusion criteria.

Given the narrative nature of this review, formal systematic review methodologies, such as PRISMA guidelines or risk-of-bias assessment tools, were not applied.

### Scope of the review

#### Zirconia in dentistry: crystallography and stabilization

**Fig. 1:**
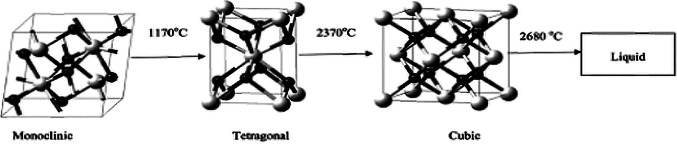
Crystallographic phase change with the variation of temperature of three ZrO_2_ phases (Adopted from Reference (Gautam et al. 2016))


Pure zirconia is a polymorphic material that theoretically exists in three crystalline forms depending on the composition and temperature. These states are: monoclinic at room temperature, tetragonal above ~ 1,170 °C, and cubic above ~ 2,370 °C (Khamverdi and Moshiri 2013) (Fig. 1). Each transformation phase has specific mechanical and optical properties. Monolithic zirconia is typically present in its most stable form, a monoclinic phase, at room temperature. However, this phase exhibits low mechanical properties, which need to be strengthened through a firing procedure to enable its use in high-stress areas. Thus, a transformation from a monoclinic phase to a tetragonal phase or cubic phase is achieved by incorporating dopants, such as yttrium, into the powder, which allows the cubic crystal structure to be partially stabilized within the microstructure at room temperature; therefore, the zirconia is strengthened (Guth et al. 2019; Wang et al. 2022). Zirconia phase transformation can negatively impact the mechanical properties of this material (Öztürk and Can 2019).

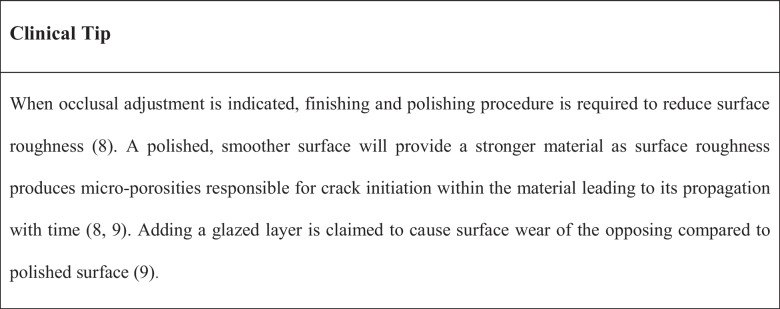


Zirconia is known to be an opaque material, which prevents its use in the esthetic region. Several strategies have been proposed to overcome this optical deficiency. Increasing the zirconia grain size will enhance the contrast ratio by promoting better light diffusion transmission and reducing light scattering within the material. In contrast, this mechanism negatively affects the zirconia strength. Low-temperature degradation (LTD) is a kinetic process that requires a moist environment and results from the spontaneous transformation of the tetragonal phase to the monoclinic phase (Chevalier et al. 2009; Ghodsi and Jafarian 2018). This phenomenon is more pronounced when the zirconia grain size exceeds 1 µm, leading to reduced structural stability and compromised optical properties (Khamverdi and Moshiri 2013). However, reducing the grain size will result in a high in-line transmission, which will enhance zirconia translucency. A grain size between 77 and 70 nm is recommended to produce a more translucent zirconia (Ghodsi and Jafarian 2018). Finally, increasing yttria content will lead to an increase in the cubic phase of zirconia and a decrease in the tetragonal phase, promoting translucency (Ghodsi and Jafarian 2018).

Multiple elements are added to the zirconia structure, including yttrium (Y), calcium (Ca), cerium (Ce), and magnesium (Mg), among others, to establish a stable form of the material at room temperature (Kongkiatkamon et al. 2023). Among which yttria has proven to be an absolute powerhouse in terms of both toughness and strength (Zhang and Lawn 2018). The addition of yttria is beneficial to the zirconia material, as it reduces grain growth, improves thermal stability, and stabilizes the tetragonal form, resulting in partially stabilized zirconia (Kongkiatkamon et al. 2023). Research established 12 types of yttrium-based zirconia structures, classified according to yttria loading: 3Y-TZP, 4Y-TZP, 5Y-TZP, and 6Y-TZP with decreasing mechanical properties, respectively. Also, it is categorized based on yttria-based zirconia layers (multilayer) (Kongkiatkamon et al. 2023).
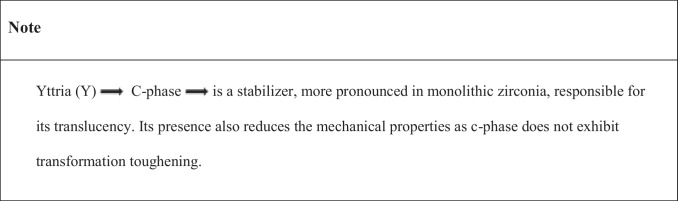


Zirconia material can be exclusively processed by computer-aided design/computer-assisted manufacture (CAD/CAM) machines or copy milling units. Zirconia blocks are supplied in two forms, either in the white state (soft chalk-like state) or the sintered state (Stawarczyk et al. 2017). Soft chalk-like or green-state zirconia blocks are easy to mill but require sintering after milling to achieve their maximum strength. Shrinkage is an inevitable result of sintering; therefore, the milling machine should be calibrated to compensate for the expected dimensional change. This method offers a short milling time, facilitating easier processing and higher productivity (Oh et al. 2010). However, sintered State Zirconia Blocks are dense blocks that are milled to the final restoration dimension, as they do not require heat treatment afterwards. It exhibits high strength, a lower pore volume, and enhanced resistance to hydrothermal aging. The major disadvantage of this material is long milling time and rapid wear of milling tools (Oh et al. 2010). Lastly, chairside or “Fast sintering” Zirconia: these materials were marketed to facilitate chairside sintering by reducing the sintering time to 20 min, instead of the traditional 8 h, through the use of special speed-sintering furnaces (Sulaiman 2020).

#### Generations of dental zirconia

Monolithic zirconia is considered a subtype of dental ceramic, and due to its excellent mechanical properties, acceptable esthetic performance, low corrosion potential, and good chemical properties, this material is the material of choice for extensive indirect dental restorations in clinical practice, especially when restoring multiple missing posterior teeth (Kontonasaki et al. 2019).

Recently, high translucence monolithic yttria-partially stabilized zirconia (HTY-PSZ) was introduced to the market. This material contains a high percentage of yttrium oxide (Y_2_O_3_), allowing a more translucent zirconia. The microscopic structure of this material is composed of a significant amount of the cubic phase (c-phase) with a tightly closed packing of numerous minute grains. This phase plays an important role in improving the translucency of the zirconia. However, the yttria component decreases the fracture strength and toughness; this is because the c-phase does not exhibit the transformation toughening phenomenon, hence limiting its use to anterior esthetic areas (Juntavee et al. 2021).

Zirconia material can be subdivided into four generations depending on the polycrystalline and yttria-stabilized dental zirconia that determine the optical and mechanical properties of these materials (Güth et al. 2019). A more recent review classified yttria-stabilized dental zirconia into three categories: Monochromatic and uniform composition, polychromatic multilayer and uniform composition, and polychromatic multilayer and hybrid composition. Each category has several sub-categories with different physical and optical properties (Kongkiatkamon et al. 2023).

#### Zirconia generations from veneering to monolithic:

A. First generation: 3-mol%-yttria-stabilized tetragonal zirconia polycrystals (3Y-TZP)

**Fig. 2:**
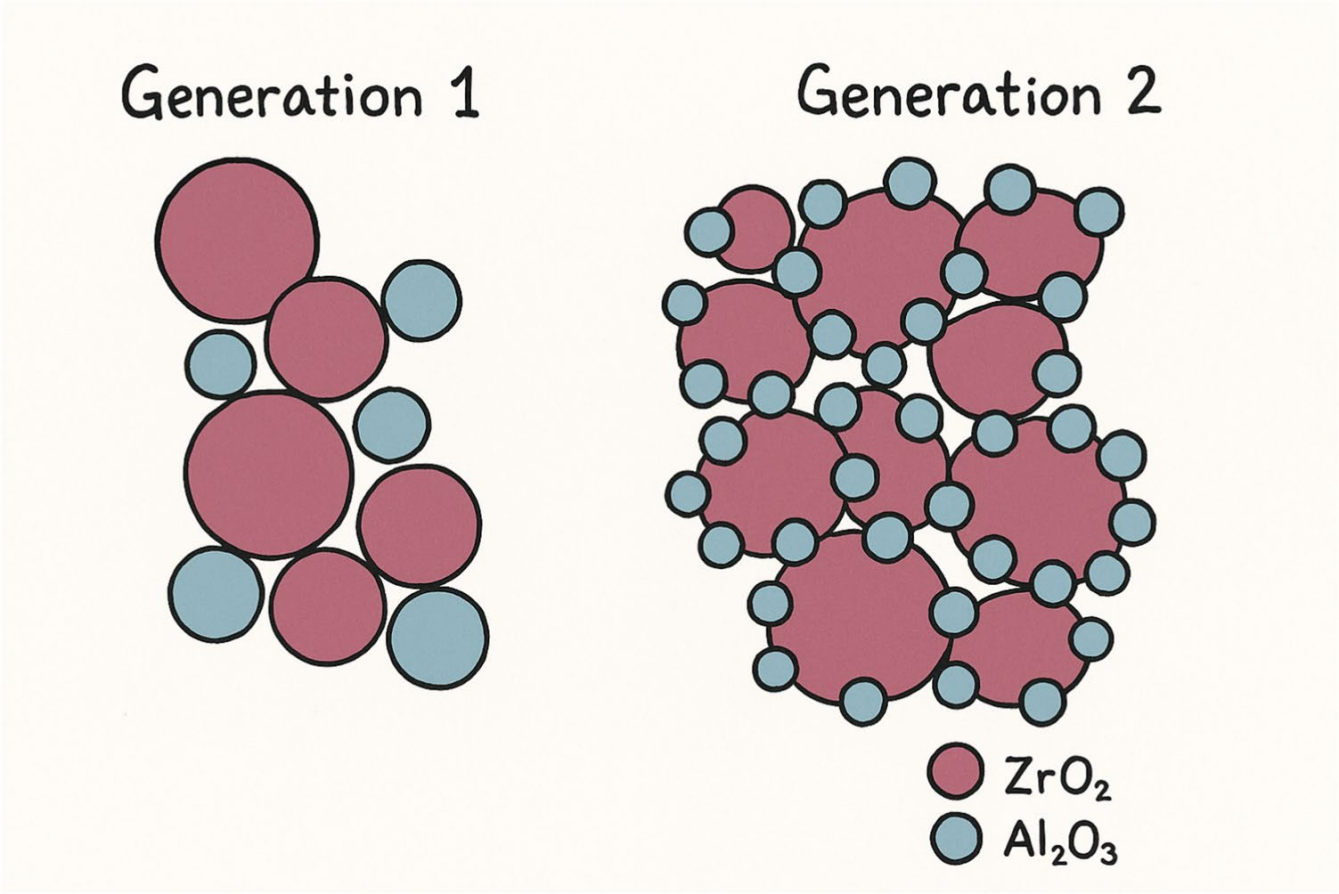
Illustration of first- and second-generation zirconia (Adopted from Reference (Stawarczyk et al. 2017))

It is a partially stabilized zirconia in the tetragonal phase (Fig. 2), which exhibits the highest mechanical properties, making it suitable for use as a framework material. This type of material is contraindicated in the fabrication of monolithic zirconia because of its high opacity (Güth et al. 2019).

B. Second generation: 3-mol%-yttria-stabilized tetragonal zirconia polycrystals with reduced alumina content (3Y-TZP)

In this category, the alumina grain (Al2O3) component in the zirconia was reduced in number and size (Fig. 2). Furthermore, alumina grains were added at the boundaries of zirconia grains, hence allowing for better light transmission, which plays a major role in the material's translucency (Güth et al. 2019). This generation has good strength and better optical properties that allow the material to be used as framework material for one and multi-unit fixed dental prosthesis FDP (Güth et al. 2019).

C. Third generation: 5-mol%-yttria-stabilized tetragonal zirconia polycrystals (5Y-TZP)

This generation is described as a fully stabilized zirconia with a cubic-tetragonal microstructure, comprising 50% cubic zirconia (Stawarczyk et al. 2017). In this category, an increased yttria content of 5 mol% is achieved. The mechanism behind its translucent appearance is that the cubic crystals are larger than the tetragonal crystals, allowing light to pass through fewer boundaries and porosities (Güth et al. 2019). This material is considered a possible alternative to high-strength glass ceramics, enabling the production of single-tooth restorations and FDPs up to three units (Güth et al. 2019).

D. Fourth generation: 4-mol%-yttria-stabilized tetragonal zirconia polycrystals (4Y-TZP)

When compared to previous generations, this generation reduced the yttria content to 4 mol%, which enhances the mechanical properties; however, it is accompanied by a reduction in its optical properties. Its main indication is short-span multi-unit FDPs (Güth et al. 2019).

## Thematic sections

### Mechanical properties of monolithic zirconia

**Fig. 3:**
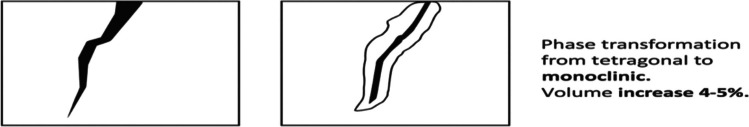
The behavior of zirconia at crack edges (Adopted from Reference (Stawarczyk et al. 2017)

A special characteristic of zirconia is its high fracture toughness, which is evident by its ability to hinder crack propagation at its tip. When tensile stress is concentrated within the tetragonal phase of zirconia, this stress converts the crystal from the tetragonal “t” to the monoclinic “m” phase (Fig. 3). A volume expansion of the crystals is observed, causing a favorable compressive stress. This mechanism is known as transformation toughening, which enables the zirconia structure to exhibit high flexural strength and fracture toughness. Furthermore, this process is reversed if the zirconia material is subjected to high temperature in a dry atmosphere (Khamverdi and Moshiri 2013; Chevalier et al. 2009).

Flexural strength of zirconia is primarily related to the structural composition. In high-translucency monolithic zirconia, an increase in the percentage of yttria leads to an increase in the cubic phase. Subsequently, decreasing the tetragonal phase, which is responsible for the transformation toughening phenomenon. Thus, the c-phase allows a crack to propagate, thereby dramatically minimizing zirconia strength and leading to low fracture resistance (Sulaiman 2020).

Flexural strength of monolithic zirconia ranges from 608 to 1540 MPa, and these differences mainly occur due to the sintering parameters, microstructure, and surface treatment (Öztürk and Can 2019). Using material with high mechanical properties is generally recommended. However, this transformation is accompanied by a shrinkage of up to 4.4% by volume. Stabilizers should be added to the composition to delay the creation of the backward phase transformation. Consequently, the zirconia in their tetragonal “metastable” (Oh et al. 2010) and cubic form are maintained at room temperature (Khamverdi and Moshiri 2013).

The mechanical and structural properties of monolithic zirconia are primarily influenced by several factors, including surface treatments, sintering parameters, and microstructure. Some claimed that an increase in sintering parameters will result in a decrease in the flexural strength of zirconia, leading to a reduction in its mechanical properties (Kilinc and Sanal 2021). On the other hand, another author stated that sintering parameters do not affect the flexural strength of zirconia ceramics (Öztürk and Can 2019).

Additionally, the thickness of restorations is one of the key factors influencing flexural strength. Generally, dental ceramics require a sufficient thickness to withstand fracture. The recommended thickness should range between 0.5 and 1 mm for all restoration margins with no sharp angles to reduce stress concentration, which initiates fracture. Other studies have suggested a more practical range of 1.0 to 1.5 mm for clinical convenience (Juntavee et al. 2021).

### Surface treatment strategies for monolithic zirconia

The long-term survival and clinical success are mainly dependent on the effectiveness of the bond strength between the zirconia ceramics and the resin cement used for cementation (Menani et al. 2014; El-Korashy and El-Refai 2014). Due to the polycrystalline structure of zirconia, multiple methods have been proposed to overcome this problem, including plasma spraying, silica coating, selective etching technique, laser, and air-borne particle abrasion using alumina (Al_2_O_3_) or other materials (Quigley et al. 2021).

#### Air abrasion

Air-abrasion is the commonly used protocol for zirconia surface treatment prior to cementation. It is to mechanically treat the intaglio surface of the zirconia restorations by the application of airborne-particle abrasion with ≤ 50 mm alumina particles at 2 bar pressure. Air abrasion is affected by several variables, including abrasive grain size, spraying distance, and angle (Kim et al. 2022). Changing both sandblasting distance and angle can negatively affect the surface roughness of the zirconia, resulting in the reduction of bond strength. However, these variables do not affect the biaxial flexural strength (Zeighami et al. 2017). On the other hand, some studies have shown that airborne particle abrasion increases the flexural and bond strength of the zirconia material (Hjerppe et al. 2016). Moreover, other authors have stated that sandblasting with alumina particles has a significant effect on phase transformation, which contributes to a higher flexural strength. Regardless of abrasion parameters, abrasion improved the flexural strength by increasing the phase transformation (Aurélio et al. 2016).

Regarding the particle size of alumina, larger-sized particles did not influence the shear bond strength of resin-ceramic adhesion; however, it resulted in a different morphological pattern after sandblasting (Sciasci et al. 2015). Mechanical surface treatment generates irregularities that promote micromechanical interlocking for resin cements (Kim et al. 2022). One researcher stated that air-abrasion and sandblasting have an influential effect that promotes surface damage in the form of sharp scratches and grooves, particularly when alumina particles with a size of 50 µm are used, which may act as stress concentration sites and reduce fracture resistance (Aboushelib 2012). Although sandblasting has a positive contribution to bonding, it may alter the zirconia surface structure due to the applied stresses, resulting in a tetragonal-to-monoclinic phase transformation on the zirconia surface (Youssef et al. 2023). Following the surface treatment, the application of ceramic primer containing the 10-methacryloyloxydecyl dihydrogen phosphate monomer (10-MDP) allows a true chemical reaction between the hydroxyl groups of zirconia and the phosphate ester monomer of the 10-MDP-containing agent (Akay et al. 2017). However, the main problem facing 10-MDP is its susceptibility to hydrolytic degradation (Sulaiman 2020; Kim et al. 2022).

#### Chemical conditioning

Chemical conditioning with acids is not beneficial for zirconia; the application of hydrofluoric acid (HF) does not produce a significantly roughened surface that is necessary for micro-mechanical retention (Menani et al. 2014). However, some experimental studies recommended hot etching as a promising approach for zirconia surface treatment (El-Korashy and El-Refai 2014). Hot acid surface treatment can be either a 40% HF solution at room temperature or a 20% HF etching solution at 70–80 °C (Kim et al. 2020). One study found that combining hot acid etching with an acidic adhesive monomer-containing primer improved both initial and long-term bond durability compared to the alumina sandblasting technique (Xie et al. 2013). When considering hot acid etching, a 60-min application duration showed higher immediate and aged shear bond strength compared to 10- and 30-min applications. Additionally, 60 min of hot acid etching application showed a higher shear bond strength than sandblasting with alumina (Lv et al. 2023). In another study, both surface treatment protocols exhibited phase transformation; however, surface treatment with hot acid etching for 10 min resulted in the lowest monoclinic phase transformation (Kang et al. 2020). Additionally, some researchers have reported that micromechanical and chemical conditioning surface treatments can induce microcracks, which are believed to decrease the flexural strength of zirconia (Kim et al. 2022).

#### Light amplification by stimulated emission of radiation

Light Amplification by Stimulated Emission of Radiation (LASER) is another technique proposed in the literature. It is the use of a laser for surface treatment, such as an Erbium, Chromium: Yttrium-Scandium-Gallium-Garnet (Er,Cr:YSGG) laser, irradiating at power outputs of 1 W, 2 W, 3 W, 4 W, 5 W, and 6W. Surface treatment with lasers increases surface roughness, which is believed to enhance the micromechanical interlocking of a resin composite luting system to the zirconia surface (Sun et al. 2015). More predictable mechanical properties were obtained by using Er,Cr:YSGG, which resulted in less surface changes and phase transformation compared to other laser types (Yahyazadehfar et al. 2021). Neodymium-doped yttrium–aluminum-garnet (Nd: YAG), erbium-doped yttrium–aluminum-garnet (Er: YAG), and carbon dioxide (CO2) are other types of lasers used to treat the surface of zirconia. Among all laser treatments, the Nd:YAG laser recorded the highest surface roughness and wettability for zirconia materials, allowing for a high value of shear bond strength (Akar et al. 2021). Another research suggested that laser treatment could induce phase transformation by the action of high laser power and uncontrolled temperature (Akar et al. 2021).

#### Glass infiltration

Glass infiltration is a technique suggested in the literature. Studies have claimed that zirconia surfaces modified by glass infiltration have a positive, influential effect on both aesthetic and mechanical outcomes, achieved by decreasing opacity and increasing both fracture resistance and flexural strength of the final prosthesis (Mohit et al. 2022).

#### Fusion sputtering

Fusion sputtering is a protocol established by Aboushelib in 2012 to generate a rough zirconia surface by spraying an air–water jet carrying microscopic zirconia particles onto an unsintered zirconia surface, ensuring good contact and adherence. These particles fused structurally with the underlying substructure, creating undercuts that are mandated for mechanical retention with resin cements (Ali et al. 2019). It is typically depicted as a cohesive failure mode, characterized by a significantly high shear bond strength with resin cements (Ali et al. 2019). This particle layer is a controlled layer with a thickness range of 4 to 12 µm; this fine thickness is designed to prevent the risk of framework unseating and damage (Aboushelib 2012).

### Adhesive strategies and resin cements

Pre-treatment of the zirconia intaglio surface is necessary prior to bonding to promote micromechanical interlocking, which is critical for durable adhesion. Establishing a stable adhesive layer between the zirconia and resin cement increases resistance to environmental degradation. Multiple studies have recommended the use of zirconia primers to further enhance adhesion between these surfaces (Sokolowski et al. 2023).

Various zirconia primers were established to enhance the adhesive strength between the zirconia surface and the resin layer used for cementation. MDP-based primers on the zirconia surface and phosphate monomers facilitate chemical adhesion (Yue et al. 2019).

Most commonly used zirconia primers contain 10-MPD (10- methacryloyloxydecyl dihydrogen phosphate), which is claimed to improve the adhesion between zirconia and resin cement (Lee et al. 2024). This is a bifunctional monomer that can adhere to both surfaces: the zirconia surface (via its hydroxyl group) and to resin cement (via its carboxylic acid group) (Zakavi et al. 2019).

Traditionally, zinc-phosphate cements were the first choice for cements when restoring metal indirect restorations. In the early 1970 s, polycarboxylate and glass-ionomer cements began to appear on the market. In 2002, Hecht and Ludstech developed a new approach, the evolution of adhesive resin cements (Maletin et al. 2023).

The composition of resin cements is similar to that of resin composite filling materials, consisting of dimethacrylate resin monomers and inorganic fillers that are usually coated with organic silanes, promoting true chemical adhesion between these two dissimilar counterparts (Maletin et al. 2023; Sokołowski et al. 2018). Main monomers are Bisphenol-A-glycidyl methacrylate (Bis-GMA), Bisphenol-A-ethoxy dimethacrylate (Bis-EMA), Urethane dimethacrylate (UDMA), which gives the structure mechanical properties, and Triethylene glycol dimethacrylate (TEGDMA), which acts as a diluent to reduce material viscosity (Maletin et al. 2023). Incorporation of filler particles enhances the performance of resin cements and provides material strengthening. Examples of these fillers: quartz, barium silicate, strontium silicate, and ytterbium trifluoride (Maletin et al. 2023).

The recommended luting cement for zirconia is self-adhesive or resin-modified cements. Dual-cure cements are preferred for cementing ceramics that are 2 mm thick or more. The justification behind this is related to the thickness of the ceramic, which can compromise the light-curing unit's ability to polymerize the cement (Sulaiman 2020). Resin cements containing 10-MDP are preferred because the phosphate ester monomer of 10-MDP chemically bonds with the hydroxyl groups of zirconia ceramics (Kim et al. 2022). The use of a cleaning paste after the try-in step is required to effectively clean the intaglio surface of the zirconia. Hence, enhancing the bond strength (Sulaiman 2020). Some studies have shown that high-strength ceramics achieve higher success rates with resin-based cements or self-adhesive resins (Blatz et al. 2018).

Resin cements are classified according to their curing mode into light-cure or “veneer” cements, dual-cure, and chemical “self” cure. Light-cured resin cement is used with low-thickness indirect restorations < 2 mm, which ensures sufficient light is transmitted, allowing proper polymerization of the resin cements (Maravić et al. 2023). This category undoubtedly provides longevity to the resin-dentin bond, as the substrate is mostly composed of enamel (Maravić et al. 2023). It contains a photo-initiator system, comprising camphor-quinone and an aliphatic amine, which allows for demand setting, color stability, and a more efficient degree of conversion (Maravić et al. 2023). Dual- and chemical-cure resin cements are usually presented in a two-paste system, indicated to prevent premature polymerization. These two systems contain initiators, such as aromatic amines and benzoyl peroxide, which make them more susceptible to color change over time (Maravić et al. 2023).
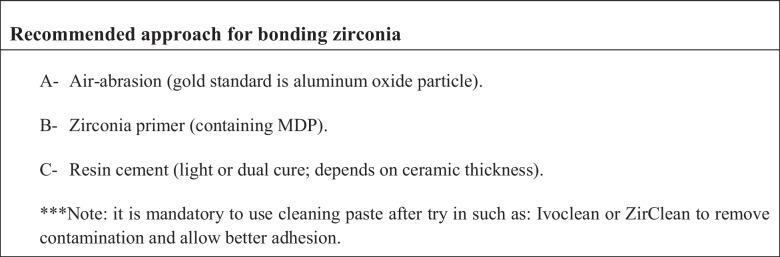


## Discussion

In clinical practice, monolithic zirconia has gained widespread acceptance because it combines favorable strength with improved esthetic outcomes. Nevertheless, the long-term success of zirconia restorations is strongly influenced by material composition, surface conditioning, and exposure to aging conditions. Evidence consistently indicates that increasing yttria content enhances translucency but is accompanied by a reduction in fracture resistance, which may compromise performance in restorations subjected to high occlusal forces, particularly in posterior regions (Zhang and Lawn 2018; Denry and Kelly 2008; Muñoz et al. 2017; Kern 2015) (49–52). Accordingly, zirconia selection should be guided by functional demands rather than esthetic considerations alone.

From a clinical bonding perspective, zirconia continues to present inherent challenges. Its polycrystalline structure and lack of a glassy phase preclude the use of conventional acid etching techniques, necessitating alternative surface treatment strategies (Blatz et al. 2003). Airborne-particle abrasion remains the most widely recommended method to improve micromechanical retention; however, excessive surface alteration may introduce surface defects and negatively affect the mechanical integrity of high-translucency zirconia materials (Kosmač et al. 1999). Although laser-based surface treatments have been proposed as less invasive alternatives, their reported effectiveness remains inconsistent and appears highly dependent on both laser parameters and zirconia composition (Akın et al. 2011; Ghoveizi et al. 2021).

Aging phenomena further influence the clinical behavior of monolithic zirconia. Thermal cycling and moisture exposure have been shown to induce microstructural changes that may reduce flexural strength and compromise bonding durability over time (Tian et al. 2025). While zirconia materials with higher cubic content demonstrate reduced susceptibility to low-temperature degradation, their limited surface reactivity may still adversely affect adhesive stability (Tian et al. 2025). Clinically, these findings emphasize the importance of conservative surface treatment approaches and strict adherence to manufacturer-recommended bonding protocols. Moreover, the variability in experimental designs and aging protocols among available studies highlights the need for standardized testing methods and long-term clinical investigations to support evidence-based restorative decision-making.

## Conclusion

Monolithic zirconia is a popular choice for dental restorations because of its strength, toughness, and biocompatibility. Improvements in yttria-stabilized zirconia now allow it to be used in both anterior and posterior regions where aesthetics is a concern. However, it can be challenging to strike a balance between mechanical properties and aesthetic demands. The material’s properties depend on the microstructure, processing method, and the thickness of the restoration. Careful finishing and polishing are crucial to prevent defects and ensure the restoration lasts.

Bonding to zirconia remains a challenge due to its unique structure. Typically, dentists use sandblasting, followed by the application of special primers containing MDP and resin cements. Other techniques, such as using lasers, hot acid, or fusion sputtering, appear promising in the lab. Cleaning the surface with special agents after attempting restoration also helps make the bond stronger and more reliable.

More long-term research and innovative surface treatments can help improve bond durability, prevent breakdown over time, and broaden the applications of zirconia in various dental cases.

## Future prospectives

Ongoing research continues to explore novel surface treatments and adhesive strategies aimed at improving bond durability and resistance to hydrothermal aging. Advances in material composition and processing techniques are expected to further expand the clinical indications of monolithic zirconia.

## Data Availability

Data sharing is not applicable to this article as no new data were generated or analyzed in this study.

## Abbreviations

*CAD/CAM*: Computer Aided Design/Computer Assisted Manufacture
*LTD*: Low Temperature Degradation
*LASER*: Light Amplification by Stimulated Emission of Radiation
*Y-TZP*: Yttria-stabilized Tetragonal Zirconia Polycrystals
*HF*: Hydrofluoric Acid
*Er,Cr:YSGG*: Erbium, Chromium: Yttrium-Scandium-Gallium-Garnet
*Nd: YAG*: Neodymium-doped Yttrium–Aluminum-Garnet
*Er: YAG*: Erbium-doped Yttrium–Aluminum-Garnet
*CO*_2_: Carbon Dioxide
*MDP*: Methacryloyloxydecyl Dihydrogen Phosphate
*Bis-GMA*: Bisphenol-A-glycidyl methacrylate
*Bis-EMA*: Bishenol-A-ethoxy Dimethacrylate
*UDMA*: Urethane Dimethacrylate
*TEGDMA*: Triethylene Glycol Dimethacrylate
